# Transient and sustained afterdepolarizations in accessory olfactory bulb mitral cells are mediated by distinct mechanisms that are differentially regulated by neuromodulators

**DOI:** 10.3389/fncel.2014.00432

**Published:** 2015-01-14

**Authors:** Guy Shpak, Asaph Zylbertal, Shlomo Wagner

**Affiliations:** ^1^Department of Psychiatry, Erasmus University Medical Center (Erasmus MC)Rotterdam, Netherlands; ^2^Sagol Department of Neurobiology, University of HaifaHaifa, Israel; ^3^Department of Neurobiology, Institute for Life Sciences, Edmond and Lily Safra Center for Brain Sciences, Hebrew UniversityJerusalem, Israel

**Keywords:** accessory olfactory bulb, vomeronasal system, electrophysiological properties, neuromodulators, carbachol, depolarizing afterpotential, hyperpolarizing afterpotential

## Abstract

Social interactions between mammalian conspecifics rely heavily on molecular communication via the main and accessory olfactory systems. These two chemosensory systems show high similarity in the organization of information flow along their early stages: social chemical cues are detected by the sensory neurons of the main olfactory epithelium and the vomeronasal organ. These neurons then convey sensory information to the main (MOB) and accessory (AOB) olfactory bulbs, respectively, where they synapse upon mitral cells that project to higher brain areas. Yet, the functional difference between these two chemosensory systems remains unclear. We have previously shown that MOB and AOB mitral cells exhibit very distinct intrinsic biophysical properties leading to different types of information processing. Specifically, we found that unlike MOB mitral cells, AOB neurons display persistent firing responses to strong stimuli. These prolonged responses are mediated by long-lasting calcium-activated non-selective cationic current (Ican). In the current study we further examined the firing characteristics of these cells and their modulation by several neuromodulators. We found that AOB mitral cells display transient depolarizing afterpotentials (DAPs) following moderate firing. These DAPs are not found in MOB mitral cells that show instead robust hyperpolarizing afterpotentials. Unlike Ican, the DAPs of AOB mitral cells are activated by low levels of intracellular calcium and are relatively insensitive to flufenamic acid. Moreover, the cholinergic agonist carbachol exerts opposite effects on the persistent firing and DAPs of AOB mitral cells. We conclude that these phenomena are mediated by distinct biophysical mechanisms that may serve to mediate different types of information processing in the AOB at distinct brain states.

## Introduction

Survival and success of animals in nature depend on their ability to communicate and create various types of social interactions with other individuals of the same species. Among all of the senses used for communication in the animal kingdom, olfaction was found to be the most widespread and conserved, and is commonly used for social interactions between mammals (Swaney and Keverne, 2009). Social communication by olfaction is mediated via semiochemicals, volatile and nonvolatile molecules, including pheromones, which are released by one individual and carry social information to receiving individuals (Regnier, 1971). Semiochemicals are detected by a number of olfactory subsystems in the nasal cavity (Munger et al., 2009), of which the best studied are the main olfactory system (MOS) and the accessory olfactory system (AOS, also known as the vomeronasal system) (Dulac and Torello, 2003). The sensory inputs to the MOS and AOS originate from sensory neurons which reside in the main olfactory epithelium (MOE) and the vomeronasal organ (VNO), and project to the main olfactory bulb (MOB) and the accessory olfactory bulb (AOB), respectively (Dulac and Wagner, 2006). The sensory terminals synapse on the principal neurons of the bulbs, the mitral and tufted cells, which are their only outputs (Mori et al., 1999).

Traditionally, the MOS was thought to function as a general odor analyzer that detects and processes volatile stimuli, while the AOS responds to a set of nonvolatile social stimuli, mostly pheromones. However, recent studies suggest the MOS is also responsive to pheromones (Stowers and Marton, 2005; Baum, 2012), while olfactory receptors that are known to be expressed in the MOE are also found in VNO neurons (Levai et al., 2006). It is now thought that the mammalian MOS and AOS detect at least partially overlapping sets of semiochemicals (Trinh and Storm, 2003; Spehr et al., 2006). Yet, the functional difference between these two chemosensory systems remains unclear.

We previously hypothesized that a main distinction between the MOS and AOS is the type of sensory information processing that takes place in the MOB and AOB (Dulac and Wagner, 2006). Accordingly, we showed that MOB and AOB mitral cells markedly differ in their intrinsic biophysical properties (Zibman et al., 2011). Most importantly, we found that unlike MOB mitral cells, AOB neurons display sustained firing responses to strong stimuli. We found these responses to be mediated by flufenamic-acid (FFA)-sensitive, calcium-activated non-selective cationic current (Ican), carried mainly by TRPM4 channels (Shpak et al., 2012).

Sustained firing responses were documented in several other brain systems, including in hypothalamic neurosecretory cells, such as the GnRH-releasing neurons (Kuehl-Kovarik et al., 2002) and the magnocellular vasopressinergic neurons of the supraoptic nucleus (SON) (Sabatier et al., 2004). These two types of neurons also exhibit depolarizing after-potentials (DAPs) following firing of one or several action potentials, and these DAPs are thought to play a role in their bursting characteristic (Kuehl-Kovarik et al., 2005; Teruyama and Armstrong, 2007; Macgregor and Leng, 2012). However, while the DAPs of SON neurons are FFA-sensitive, hence may be mediated by Ican (Ghamari-Langroudi and Bourque, 2002; Teruyama and Armstrong, 2007), those of the GnRH neurons are not (Wang and Kuehl-Kovarik, 2010).

Ican-mediated sustained firing responses are known to be highly sensitive to the effects of various neuromodulators (Congar et al., 1997; Viemari and Ramirez, 2006; Wang et al., 2011a). Especially documented is the positive effect of the cholinergic agonist carbachol on Ican activation, shown to be mediated by the muscarinic M1 receptor (Rahman and Berger, 2011; Yoshida et al., 2012; Yamada-Hanff and Bean, 2013). M1 activation was shown to enhance persistent firing in both granule and mitral cells of the AOB (Smith and Araneda, 2010). Other neuromodulators known to be active in the AOB are noradrenaline and dopamine (Dong et al., 2009; Matthews et al., 2013), both shown to be associated to the AOB-mediated mate recognition in mice (Brennan, 2009). Interestingly, both acetylcholine and dopamine were recently linked to the atypical social behavior of BTBR mice (Karvat and Kimchi, 2014; Squillace et al., 2014).

Here we examined whether AOB and MOB mitral cells also display DAPs, similarly to SON and GnRH neurons and if so, whether these DAPs are generated by the same mechanisms underlying the Ican-dependent sustained firing.

## Materials and methods

### Animals

C57BL/6J male mice (Harlan Laboratories, Jerusalem, Israel) were maintained in the SPF mice facilities of the University of Haifa under veterinary supervision, according to National Institutes of Health standards, with food and water *ad libitum* and lights on from 7:00 A.M. to 7:00 P.M. Eight- to twenty-week-old mice (25–35 g) were held in groups of 5–10 mice per cage. All experiments were approved by the Animal Care and Use Committee of the University of Haifa.

### Slice preparation and recordings

Mice were anesthetized (isoflurane; Abbott Laboratories, Chicago, IL, USA) and killed by cervical dislocation. Olfactory bulb slices, 300 μm thick, were prepared as previously described (Wagner et al., 2006). Coronal or sagittal planes were used for MOB slices and semi-coronal (Del Punta et al., 2002) or sagittal planes for AOB slices, with no differences in the results. Slices were equilibrated for 1–5 h in physiological solution containing the following: 125 mM NaCl, 25 mM NaHCO3, 15 mM glucose, 3 mM KCl, 2 mM CaCl2, 1.3 mM, NaH2PO4, and 1 mM MgCl2, oxygenated by bubbling through a 95% O2 and 5% CO2 mixture, pH 7.4. For electrophysiological recordings, slices were submerged in oxygenated physiological solution at room temperature in a recording chamber and perfused at a constant rate of 1–3 ml/min. In all experiments, gabazine (5 μM; Tocris Bioscience) or picrotoxin (50 μM; Sigma) were applied to the bath solution to block GABA_A_ receptors. Whole-cell recordings were performed using borosilicate pipettes filled with standard intracellular recording solution containing the following (mM): 120 K-gluconate, 14 KCl, 10 Na-gluconate, 10 HEPES, 3 MgATP, 0.5 NaGTP, and 10 phosphocreatine (5–12 MΩ). When BAPTA was used, BAPTA-tetrapotassium (Invitrogen, Carlsbad, CA, USA) was dissolved in the intracellular solution to a final concentration of 5 mM. When FFA was used, 50 μM of Flufenamic acid (FFA, Sigma) was dissolved in the physiological solution to block Ican. In the neuromodulation experiments the following modulators were bath-applied: carbachol (1 μM; Tocris bioscience) and dopamine hydrochloride (50 μM; Tocris bioscience). DNQX (20 μM; Tocris bioscience) and APV (40 μM; Sigma Aldrich) were used in the bath solution to block AMPA and NMDA receptors in several experiments.

### Electrophysiology

For electrophysiological recordings, the following setup was used: Olympus BX51WIF equipped with motorized stage and manipulators (Scientifica, Uckfield, UK), recording chamber (RC-26G; Warner Instruments), pulse generator (Master 8; A.M.P.I.) and MultiClamp 700 B amplifier (Molecular Devices). Mitral cells were visualized using infrared differential interference contrast video microscopy via a 40X water-immersion objective. Mitral neurons were identified by the location of the cell body, for MOB neurons strictly in the MOB mitral cell layer and for AOB mitral cells on the ventral side of the external plexiform layer of the AOB.

### Calcium imaging

For calcium imaging experiments, 50 μM Oregon Green BAPTA 1 (Life Technologies) was added to the patch pipette. Fluorescence signal, acquired using excitation at 480 nm and emission at 535 nm, was recorded at 25–100 Hz using a high speed camera (MiCAM Ultima, Brainvisions) while using a high power LED (Prizmatix, Givat-Shmuel, Israel) as an excitation light source.

### Data analysis

As previously described in detail (Shpak et al., 2012). In short, EPSC-like current injections had a 10 ms rise time constant and a 5 s decay time constant. All amplified signals were digitized at 2–10 kHz using a National Instruments board and acquired and analyzed using home-made written LabVIEW program (National Instruments, Austin TX, USA) and MATLAB (Mathworks, Natick, MA, USA). No junction-potential correction was made. For calculation of the DAP integral, the membrane baseline potential, calculated as the mean potential of a 200 ms interval before stimulation, was subtracted from the membrane voltage response to shift the baseline to zero. The area under the shifted voltage trace was calculated during 1 s immediately following the end of the injected square-pulse current. For calculation of the best fit of linear regression (**Figure 4B**), we calculated the coefficient of determination (R^2^, using regression function of Matlab) for two linear regressions separated by each integer value of firing rate between 10 and 20 Hz. The value that gave the higher sum of R^2^ (13 Hz) was selected for further analysis.

### Statistical analysis

For comparisons between control condition and the various experimental conditions, we used paired (FFA, carbachol and dopamine) or unpaired (BAPTA) 2-tail *t*-test, following Kolmogorov–Smirnov normality check. For the comparison of DAP integral between the conditions we compared the values observed at three distinct stimulation levels (20, 50, and 80 pA), with each cell represented by three traces in each level, and corrected the significance for multiple comparisons.

## Results

### Opposite directions of afterpotentials in AOB and MOB mitral cells

**Figure 1:**
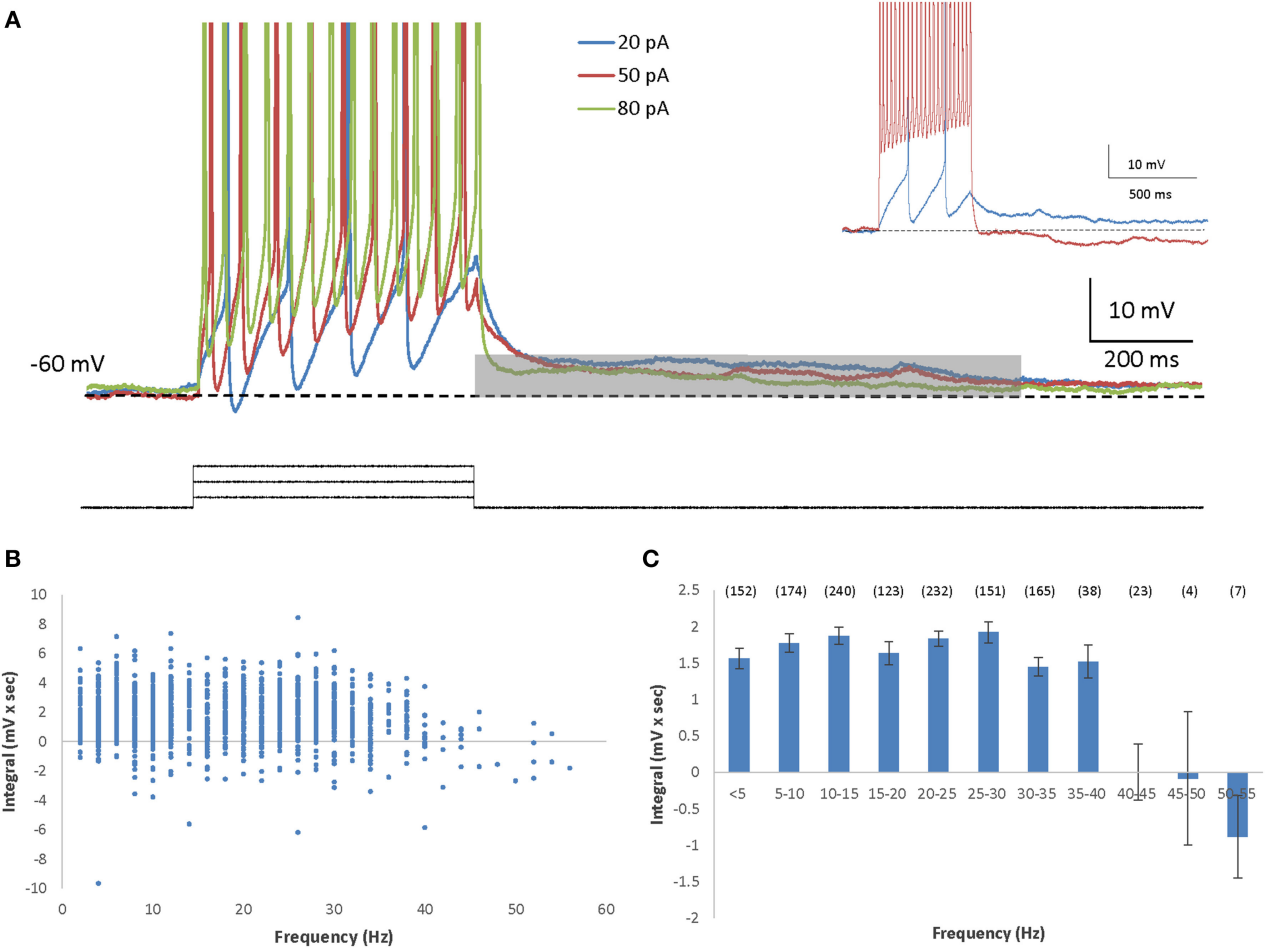
**AOB mitral cells exhibit depolarizing afterpotential (DAP). (A)** Representative superimposed voltage traces of firing responses (spikes cut at 0 mV) to three different depolarizing current steps (20, 50, and 80 pA) in the same cell (inter stimulus interval = 5 s). The DAP integral was measured by calculating the integral of the voltage traces relative the membrane baseline (dashed line), during the first second (gray bar) following the termination of the current step. Inset: Example traces from another cell showing marked ADP following low firing rate (blue) as opposed to HAP with high firing rate (red). **(B)** The DAP integral as a function of the firing frequency of 1309 stimuli given to 71 AOB mitral cells. Stimulation amplitude ranged between 5 and 150 pA. Max stimulation amplitude was determined by the saturation of the I-F response. **(C)** Mean (±s.e.m.) values of the data shown in **(B)**, using 5 Hz bins (n of each frequency in brackets above).

To stimulate firing in olfactory bulb mitral cells we used 0.5-s depolarizing square-pulse current injections of variable amplitude (5–150 pA), on top of a DC current (10–25 pA) used by us to clamp the membrane to a holding potential of −60 mV. In AOB mitral cells, this type of stimulation elicited prolonged depolarizing afterpotentials (DAPs) immediately following most levels of current steps (Figure 1A), and even following a single spike (Supplemental Figure 1A). A significant DAP that in some cases caused post-stimulation firing, could be elicited despite the presence of AMPA and NMDA receptors blockers (Supplemental Figures 1B,C), suggesting that the DAP is not caused by recurrent excitation. To quantitatively analyze these DAPs, we calculated the integral of the voltage trace relative to the baseline membrane potential during 1 s, starting at the end of the current step (Figure 1A, gray bar). When the DAP integral was plotted as a function of the mean firing frequency during the depolarizing current step, it became evident that it is relatively constant throughout most firing frequencies (Figures 1B,C). Nevertheless, beyond firing rate of 40 Hz we observed a steep reduction in the DAP integral (Figure 1A inset). The same results were observed when the DAP integral was plotted as a function of the stimulation level (Supplemental Figure 2A) and even when the cells firing above 40 Hz were analyzed separately from the others (Supplemental Figure 2B), suggesting that the DAP is a general characteristic of AOB mitral cells.

**Figure 2:**
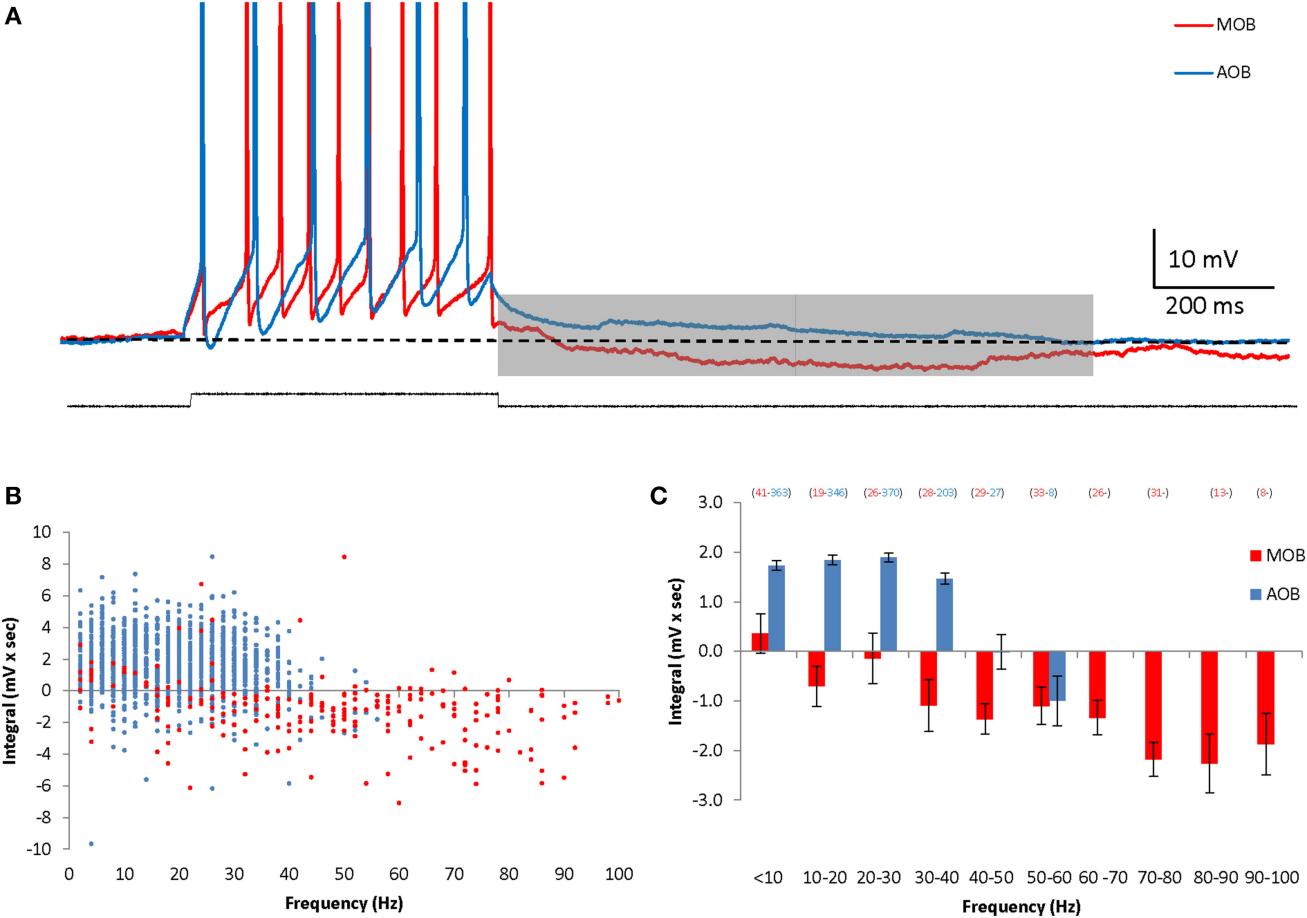
**AOB and MOB mitral cells exhibit opposite afterpotentials in response to depolarizing current injections**. **(A)** Superimposed MOB (red) and AOB (blue) voltage responses to a 30 pA square-pulse depolarizing current injection. Spikes were cut at 0 mV. **(B)** The calculated DAP integral as a function of the firing frequency in 1317 stimuli given to 70 AOB cells, and 254 stimuli given to 14 MOB cells. **(C)** Mean (±s.e.m.) values of the data shown in **(B)**, using 10 Hz bins.

In previous studies we found that MOB and AOB mitral cells show vast differences in cellular properties (Zibman et al., 2011), as well as distinct types of responses to synaptic stimulation (Shpak et al., 2012). We therefore examined here whether these two neuronal populations also differ in their membrane afterpotentials. Indeed, in contrast to the DAPs exhibited by AOB mitral cells, MOB mitral cells demonstrated robust hyperpolarizing afterpotentials (HAPs) lasting for at least 1 s following the depolarizing current step (Figure 2A). This prolonged hyperpolarization seemed to be firing frequency-dependent, with its integral gradually increasing with the increasing firing frequency (Figures 2B,C). Thus, following firing episodes, MOB and AOB mitral cells demonstrate significant afterpotentials with opposite directions that further distinguish between these two neuronal populations.

### AOB mitral cells afterpotentials are calcium dependent

**Figure 3:**
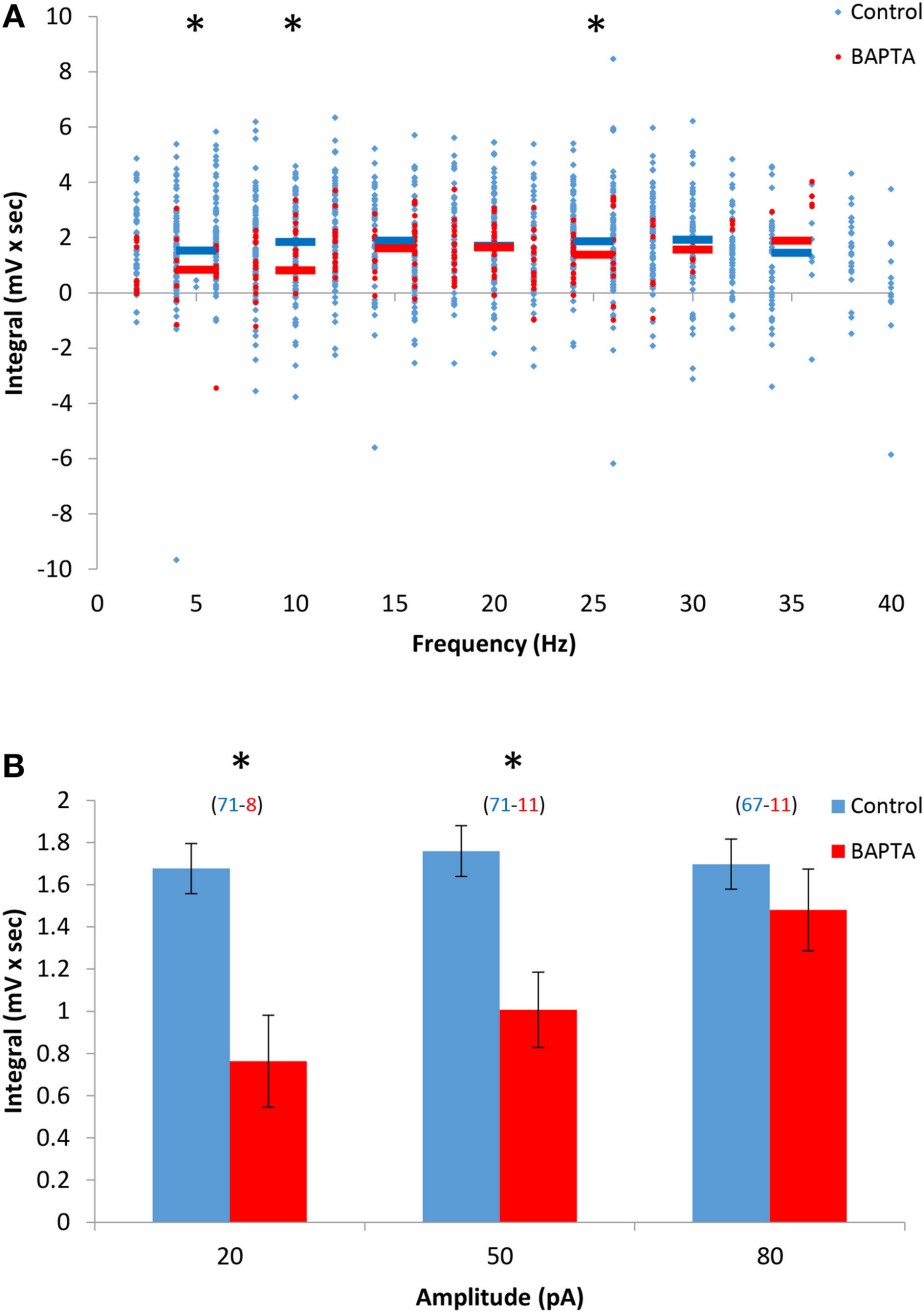
**The DAPs of AOB mitral cells depend on low levels of intracellular calcium. (A)** DAP integral of AOB mitral cells plotted as a function of firing frequency during stimulation in control (blue, *n* = 43 cells, 1345 stimuli) and 5 mM BAPTA-filled cells (green, *n* = 11 cells, 256 stimuli). Horizontal colored lined represent the mean using 5 Hz bins. **(B)** Mean (±s.e.m.) values of the responses to three current amplitudes representing low (20 pA), moderate (50 pA), and high (80 pA) stimulation levels. A significant difference (^*^p < 0.05, *t*-test corrected for multiple comparisons) was found between control and BAPTA conditions only for low and moderate stimulation levels.

We previously showed that AOB mitral cells exhibit sustained firing responses carried by a non-selective calcium-activated cationic current (Ican), which is calcium-dependent and flufenamic acid (FFA)-sensitive (Shpak et al., 2012). In order to examine the relationship between the DAPs and Ican in AOB mitral cells, we first evaluated the dependence of the DAPs on the intracellular free calcium level. We found that lowering the intracellular calcium concentration by including the calcium chelator BAPTA (5 mM) in the patch pipette caused a significant reduction of the DAPs, suggesting that the DAPs of the AOB mitral cells are indeed calcium-dependent, similarly to their sustained firing response (Figures 3A,B). However, this was the case when low levels of stimulation (up to 50 pA) were used, whereas at higher stimulation levels, when firing frequencies reached >10 Hz, no significant difference from control cells was observed. In agreement with a previous study (Ma and Lowe, 2004), we found that a significant transient calcium elevation was induced by firing of even a single spike and was made stronger with the elevation of firing frequency (Supplemental Figures 3A–C). Moreover, in the presence of BAPTA, high levels of firing frequency (10–30 Hz) induced transient calcium elevations which were comparable to those achieved with low firing frequency (1–5 Hz) in control condition (Supplemental Figure 3D). Therefore, combining these results with the almost maximal DAP integral observed in control cells already at very low firing rate (<5 Hz, Figure 1C) suggests that the DAPs depend on low levels of intracellular calcium that are reached already at very low firing rate (<5 Hz) under control conditions, and with higher firing rate (>10 Hz) in the presence of BAPTA.

**Figure 4:**
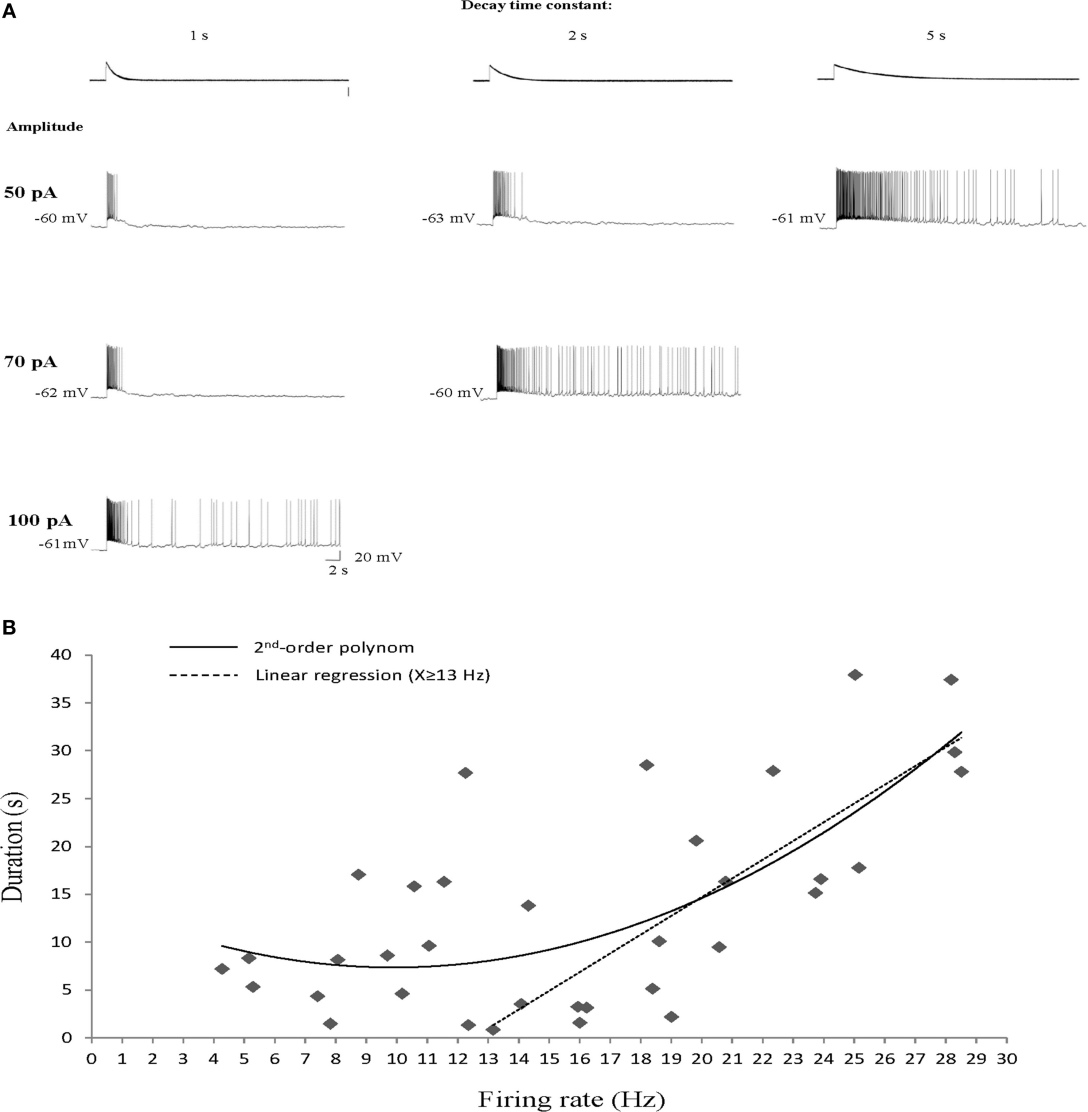
**Sustained firing responses of AOB mitral cells require intensive firing episode. (A)** Responses of a single AOB mitral cell to increasing amplitude (rows) or decay time constant (columns) of EPSC-like current injection. **(B)** Response duration plotted as a function of the mean firing rate at the first second of the response (*n* = 35 stimuli given to 8 cells of 6 animals). A 2nd-order polinom fitted the data, suggesting two types of relationships. Separating the data to two sets at firing rate of 13 Hz was found to yield the best fit of two linear regressions. Accordingly, a statistically significant correlation is observed for firing rates of ≥13 Hz (*R*^2^ = 0.6, *p* < 0.001, Spearman's) while no correlation is observed for mean firing rates of <13 Hz (*R*^2^ = 0.16, *p* > 0.2).

To further investigate this difference between the Ican and DAPs activation we characterized the dependence of AOB sustained firing responses on the initial firing rate of the response. To that end we used EPSC-like injections (Shpak et al., 2012) with variable amplitude and decay time constant. As exemplified in Figure 4A, an abrupt transition from transient to sustained firing response could be elicited in AOB mitral cells in a similar manner by either increasing the amplitude of the injected EPSC-like current or by increasing its decay time constant for a given amplitude. Both manipulations are assumed to increase the initial firing rate of the response, previously shown by us to determine the induction of the sustained firing activity (Shpak et al., 2012). We therefore plotted the duration of the firing response as a function of the firing rate during the first second of the current injection (*n* = 35 stimuli given to 8 cells of 6 animals). These data looked as if a correlation between the firing rate and response duration exists only above ~15 Hz. Indeed, a 2nd-order polynom fitted the data very well, suggesting two distinct relationships involved (Figure 4B). Assuming two linear relationships, we found that a firing rate of 13 Hz yields the best fit of two linear regressions to the data set. Accordingly, a positive correlation (*R*^2^ = 0.6, *p* < 0.001, Spearman's correlation) was observed between the response duration and the initial firing rate when the latter was ≥13 Hz (Figure 4B, dashed line), while no correlation (*R*^2^ = 0.16, *p* > 0.2) was observed below this level. Moreover, almost no sustained (>10 s) firing was observed for initial firing rate of <10 Hz. This is in sharp contrast to the DAPs that were fully activated already at firing rates of 5–10 Hz (Figure 1C). Thus, while both the DAPs and sustained firing in AOB mitral cells are calcium-dependent, they show very different sensitivities to intracellular calcium levels, with the DAPs being more sensitive, hence induced already at low firing rate, and the sustained firing being less sensitive, hence activated only following intensive firing.

### AOB mitral cells afterpotentials are insensitive to FFA

**Figure 5:**
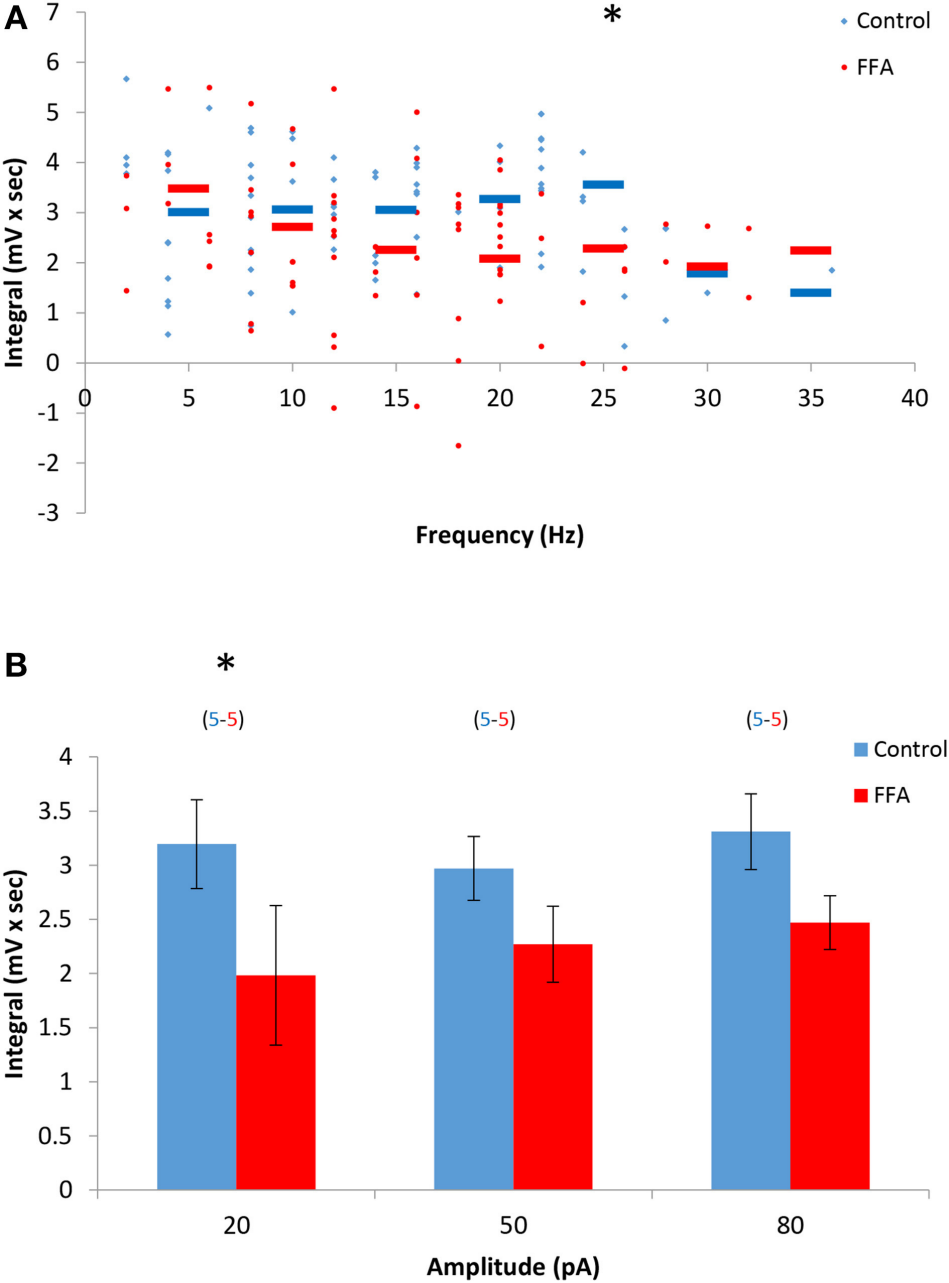
**The DAPs of AOB mitral cells are only partially sensitive to flufenamic acid (FFA)**. **(A)** DAP integral of AOB mitral cells plotted as a function of firing frequency evoked by square pulse stimulation under control conditions (blue, *n* = 5 cells, 79 stimuli) and in the presence of 50 μM FFA (green, same 5 cells, 80 stimuli). **(B)** Mean (±s.e.m.) values of the responses to three current amplitudes representing low (20 pA), moderate (50 pA), and high (80 pA) stimulation levels. A significant difference (^*^p < 0.05, paired *t*-test corrected for multiple comparisons) was found between control and BAPTA conditions only for low level of stimulation.

The marked differences in dependency of the DAPs and sustained firing responses of AOB mitral cells on intracellular calcium level suggest that they are mediated by distinct biophysical mechanisms, presumably distinct conductances. To further assess this possibility, we examined the sensitivity of the DAPs to FFA, a non-selective Ican blocker previously shown to abolish the sustained responses of AOB mitral cells (Shpak et al., 2012). We found that the DAPs were only partially blocked in the presence of FFA (50 μM), with statistically significant change only in low stimulation levels (20 pA, Figures 5A,B). These results further support the notion that distinct biophysical mechanisms are involved in the DAPs and sustained firing responses of AOB mitral cells.

To further explore this issue we examined the effects of two neuromodulators, carbachol and dopamine, known to be active in the AOB (Dong et al., 2009; Matthews et al., 2013) on the sustained firing responses and DAPs in AOB mitral cells.

### The cholinergic agonist carbachol differentially affects the various afterpotentials in AOB mitral cells

**Figure 6:**
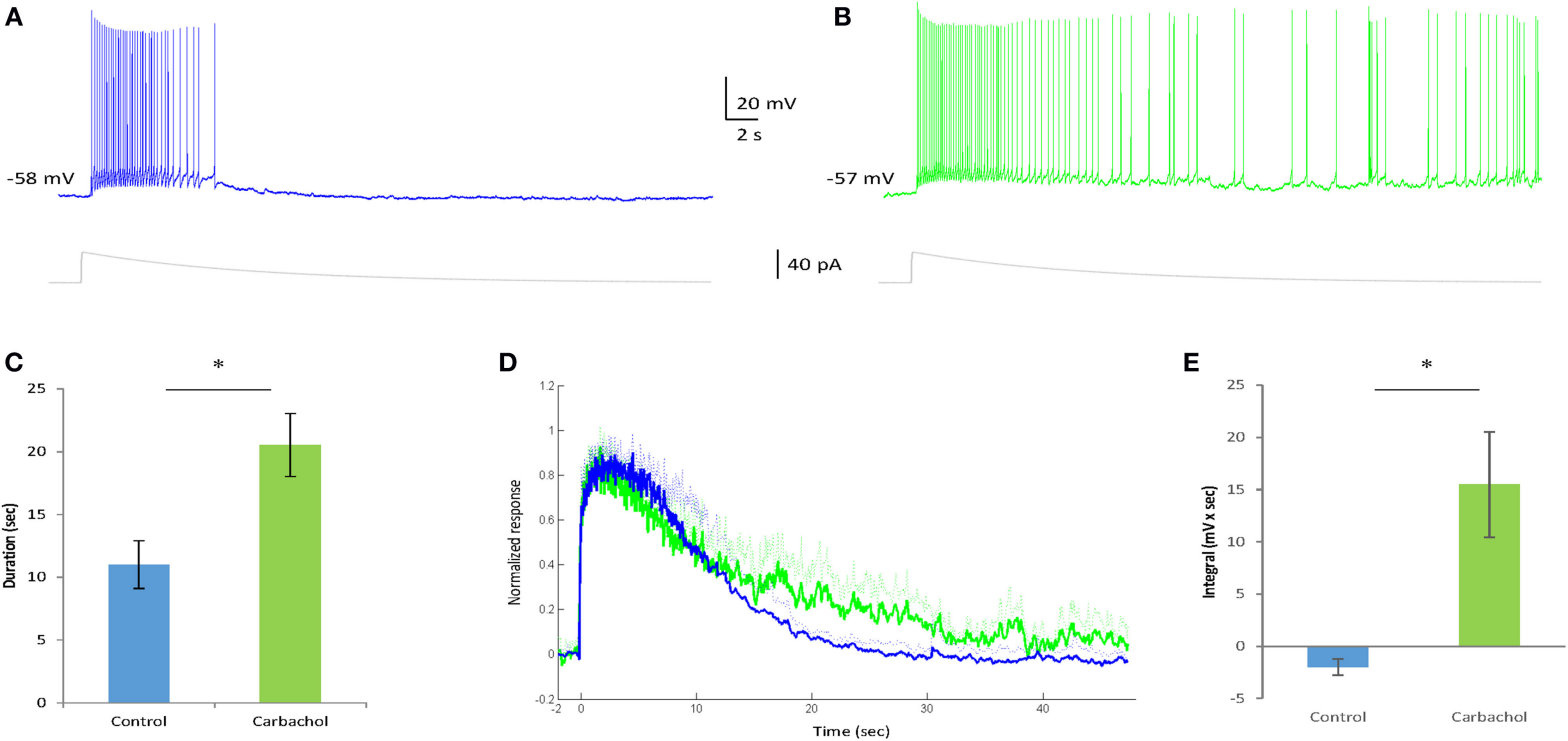
**Carbachol enhances the sustained firing response in AOB mitral cells. (A,B)** Typical voltage responses of an AOB mitral cell to injection of EPSC-like current (40 pA, gray trace below) in the absence **(A)** and presence **(B)** of Carbachol (1 μM) in the bath solution. The injected current amplitude was selected to be below the level sufficient for persistent firing induction in this cell under control condition (weak current injection). **(C)** Comparison of the firing duration of five cells (from four animals) to 20 pA EPSC-like current injection in control and carbachol conditions (^*^p < 0.05, paired *t*-test). **(D)** Mean normalized voltage trajectories of the responses of the same cells as in **(C)**, recorded before (blue) and after (green) carbachol application to the bath solution. Dashed lines represent SD values to one direction. **(E)** Comparison of the integral (20–30 s post-stimulation) under the voltage trajectories of the same cells (without normalization) between control and carbachol conditions (^*^p < 0.05, paired *t*-test).

Carbachol is well known to positively modulate Ican activation in various brain regions (Rahman and Berger, 2011; Yoshida et al., 2012; Yamada-Hanff and Bean, 2013). To test its effect on the sustained firing of AOB mitral cells we recorded their responses to relatively weak EPSC-like stimuli (below the level required for sustained firing induction under control condition) before and after application of carbachol (final concentration = 1 μM, *n* = 5 cells from 4 animals). As expected, carbachol presence promoted sustained firing in response to the weak stimuli that could generate only a transient response under control conditions (Figures 6A,B). Accordingly, the duration of the firing response was significantly increased in the presence of carbachol [from 10.1 ± 1.9 to 20.5 ± 2.5 s, *t*_(4)_ = 4.6, *p* = 0.01, Figure 6C]. Moreover, this effect was accompanied by a clear prolongation of the ramp potential underlying the firing response (Figure 6D), reflected by a significant increase of the integral of the ramp potential calculated between 20–30 s following stimulation (Figure 6E). Similar effects of augmentation were observed when higher stimulation levels were used (not shown).

**Figure 7:**
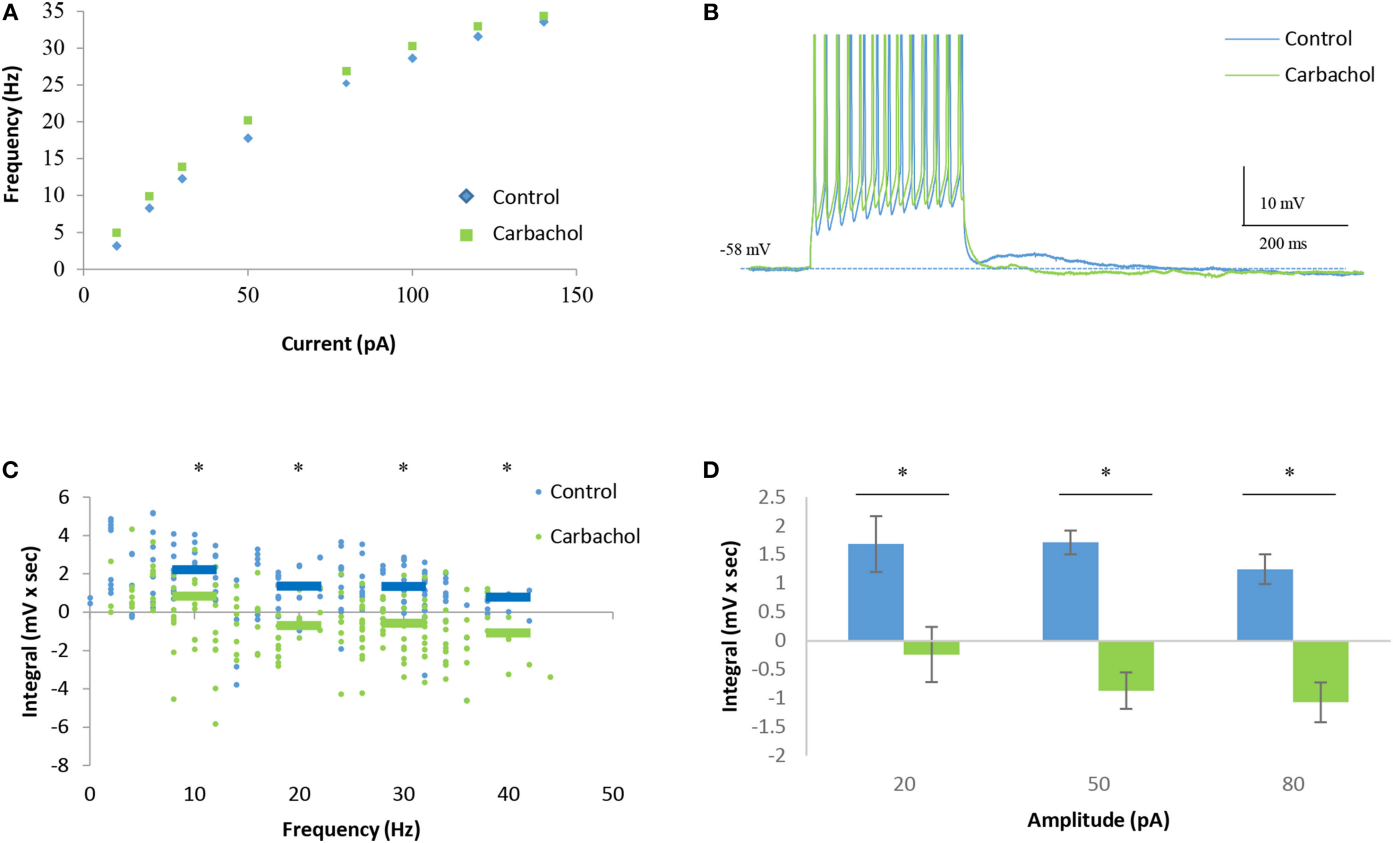
**Carbachol blocks DAPs in AOB mitral cells. (A)** Mean current-frequency (I-F) curves of the same cells as in Figure 6 before (blue) and after (green) carbachol (1 μM) application to the bath solution. **(B)** Representative voltage responses to 40 pA square-pulse stimulation of the same cell in control and carbachol conditions. Spikes were cut at 0 mV. **(C)** Mean (±s.e.m.) DAP integral of all responses of the same cells, plotted as a function of the firing frequencies. Horizontal lines represent the mean using 10 Hz bins. **(D)** Mean (±s.e.m.) values of the DAP integral of the responses to three current amplitudes representing low (20 pA), moderate (50 pA), and high (80 pA) stimulation levels. A significant difference (^*^p < 0.05, paired *t*-test corrected for multiple comparisons) was found between control and carbachol conditions for all stimulation levels.

In contrast, despite showing no effect on the IF curve of the cells (Figure 7A), carbachol caused a marked reduction in the DAPs already at low firing rates (Figures 7B,C). Moreover, at firing rates of >10 Hz carbachol caused the appearance of HAPs rather than DAPs (Figure 7C). These changes were found to be statistically significant at all stimulation levels (Figure 7D). The opposite effects of carbachol on the sustained firing response and DAPs in AOB mitral cells further supports the conclusions that they are subserved by distinct mechanisms that can be differentially modulated.

In contrast to carbachol, dopamine application (50 μM) did not cause any significant change in the firing properties examined by us (data not shown).

## Discussion

In this study we showed that, unlike the robust HAPs exhibited by MOB mitral cells, AOB mitral cells display DAPs lasting for at least 1 s following firing episodes. These DAPs, induced even by single spikes, are independent of firing frequency in the range of 5-40 Hz. The DAPs of AOB mitral cells are calcium-dependent but not FFA-sensitive, suggesting a different mechanism from the Ican activation, previously described by us in these cells (Shpak et al., 2012). Furthermore, the Ican and DAPs are differentially affected by the cholinergic agonist carbachol, which further supports their distinct biophysical basis.

Several previous studies showed that extended DAPs can be induced also in MOB mitral cells, if inhibition is blocked, and that they are mediated by NMDA receptor (NMDAR)-dependent dendrodendritic recurrent self-excitation (Aroniadou-Anderjaska et al., 1999; Friedman and Strowbridge, 2000; Didier et al., 2001; Salin et al., 2001; Maher and Westbrook, 2005). This mechanism is highly unlikely to underlie the DAPs observed by us in AOB mitral cells because of several reasons: (1) The NMDAR-dependent DAPs in MOB mitral cells can be observed only in very specific conditions where potassium currents are pharmacologically blocked and the magnesium blockade of NMDAR is removed, either by depolarization (using TTX to block sodium spikes) or by using low-magnesium solutions. None of these conditions were employed in our experiments, and indeed MOB mitral cells did not show DAPs but rather HAPs. (2) The NMDAR-dependent self-excitation depends upon synaptic vesicle release hence was shown to be highly sensitive to calcium concentration and completely blocked by the presence of BAPTA (Friedman and Strowbridge, 2000; Salin et al., 2001). In our case, DAPs seem to require low levels of free intracellular calcium hence could be elicited even in the presence of BAPTA. (3) Since the self-excitation in MOB cells is mediated by synaptic glutamate release, it is strongly graded with the number of spikes in a train (Friedman and Strowbridge, 2000). This is in contrast to the DAPs in AOB mitral cells which seems to saturate in very low firing frequency. (4) The DAPs observed by us could not be blocked by specific antagonists of AMPA and NMDA receptors (Supplemental Figures 1B,C). Therefore, we conclude that the DAPs in AOB mitral cells are elicited by intrinsic mechanism, similarly to Ican induction in these cells.

DAPs with various characteristics are known to be displayed by numerous types of neurons in the mammalian central nervous system (Major and Tank, 2004). Interestingly, hypothalamic GnRH (Kuehl-Kovarik et al., 2005) and SON vasopressinergic neurons (Teruyama and Armstrong, 2007) show DAPs that are very similar in their characteristics to those found by us in the AOB. The reason for that similarity is not clear and may be related to the role of all three neuronal populations in reproductive and social behavior. Yet, the DAPs of GnRH and SON neurons were shown to be mediated via distinct mechanisms: whereas those displayed by SON neurons are sensitive to the Ican blocker FFA, thus thought to be mediated by Ican activation (Ghamari-Langroudi and Bourque, 2002), the DAPs recorded in GnRH neurons are insensitive to FFA, hence are probably mediated by a different mechanism (Wang and Kuehl-Kovarik, 2010). Here we showed that, similarly to GnRH neurons, AOB mitral cells exhibit DAPs that cannot be blocked by FFA, and therefore are unlikely to be mediated by Ican.

The conclusion that the Ican-mediated sustained firing responses of AOB neurons and the DAPs exhibited by them depend on distinct mechanisms is further supported by their very different dependency on firing-induced calcium influx. We showed here that activation of sustained firing responses by EPSC-like current injection requires a period of intensive firing in a rate that is >15 Hz, whereas DAPs were initiated even by a single spike and reached saturation already at 5–10 Hz. These results suggest that while Ican induction depends on relatively high intracellular free calcium levels, which are reached only following intensive firing, DAP initiation requires only low calcium levels that are reached even with minimal firing. This conclusion is further supported by our data, showing that Ican and DAPs are differentially influenced by the presence of the calcium chelator BAPTA in the patch pipette: whereas Ican activation was completely blocked, suggesting activation by high calcium levels that cannot be reached with BAPTA, DAPs appear normally with BAPTA, but require higher firing rate (>10 Hz), suggesting dependency on low calcium levels that can be reached even in the presence of BAPTA, given high enough firing rate.

Persistent neural activity, defined as sustained change in action potential discharge that long outlasts a stimulus, represents a fundamental form of brain dynamics that is thought to be involved in various types of cognitive processes, including working memory, sensory information processing, and execution of motor programs (Major and Tank, 2004). These processes are known to be regulated by various neuromodulators (Yoshida et al., 2008; Arnsten, 2011; Wang et al., 2011b; Yoshida et al., 2012) that induce specific states in certain neural networks according to the internal and external contexts of the animal (Marder, 2012). Our data, showing that Ican activation and DAP induction in AOB mitral cells rely on distinct mechanisms, raised the possibility that these two types of persistent neural activity may be differentially modulated in AOB neurons by distinct neuromodulators, creating a dynamic range of sustained activity in this network. To test this possibility, we examined the effect of two neuromodulators known to be active in the AOB (Dong et al., 2009; Matthews et al., 2013) on the dynamics of firing responses in AOB mitral cells. Since our aim was to get a general idea on the susceptibility of persistent activity mechanisms in the AOB to neuromudulation, we did not thoroughly examine a wide range of concentrations for each ligand, but rather examined their influence in concentrations that were previously shown to be effective in brain slices. Whereas dopamine had no influence on both phenomena, we found a marked difference between the effects of the muscarinic agonist carbachol on Ican activation and DAP induction.

Carbachol is well known for its efficiency in promoting Ican activation in multiple brain regions, mainly via the M1 muscarinic receptor (Rahman and Berger, 2011; Yoshida et al., 2012; Yamada-Hanff and Bean, 2013). In the AOB it was shown to enhance persistent firing in both granule and mitral cells, mainly via activating muscarinic receptors (Smith and Araneda, 2010). In accordance with these results we found that carbachol causes a significant enhancement of Ican induction in AOB mitral cells. In contrast, as previously reported (Smith and Araneda, 2010), it caused replacement of the DAPs with HAPs. It is not clear whether this effect of carbachol is caused by inhibiting the DAPs, by enhancing the HAPs, or by both activities. Yet, the overall effect is elimination of this type of short-term persistent neural activity. The opposite effects of carbachol on both types of persistent neural activity in AOB mitral cells not only support their distinct biophysical bases but also show that they may be separately modulated. We suggest that in control condition, where the firing-frequency requirement for induction of Ican-mediated persistent firing is high, the presence of DAPs creates a longer time window for integration of inputs that will enable the cell to pass the threshold for persistent firing. This may reflect a “base” state, when the animal is not anticipating any specific social input. In contrast, upon cholinergic activity in the AOB, the lowering of the threshold for Ican-mediated persistent firing is accompanied by appearance of HAPs instead of DAPs, which will restrict the time window for input integration in AOB mitral cells. Thus, cholinergic activity in the olfactory bulb, linked to attentional behavioral states (D'souza and Vijayaraghavan, 2014), may reflect anticipation for a social encounter which is assumed to be associated with a lot of sensory inputs arriving within a short time window to AOB mitral cells.

Thus, by combining the distinct effects of different modulators, the activity of AOB mitral cells may adopt highly variable dynamics of persistent firing that may drive different behaviors according to the social context of the animal.

### Conflict of interest statement

The authors declare that the research was conducted in the absence of any commercial or financial relationships that could be construed as a potential conflict of interest.
