# Supplementary material for: Transient and sustained afterdepolarizations in accessory olfactory bulb mitral cells are mediated by distinct mechanisms that are differentially regulated by neuromodulators
Source: Front Cell Neurosci. 2015 Jan 14;8:432. doi: 10.3389/fncel.2014.00432 (PMC4294165; doi:10.3389/fncel.2014.00432)
Supplement: Supplementary file 1 [file DataSheet1.DOCX]

**Supplemental Fig. 1: DAP examples**

1. An example of DAP following firing of a single spike. Mean base line membrane potential is -62 mV (dashed line).
2. The mean voltage response (blue) to a step current injection (black) that evoked 4-18Hz firing in a single cell, recorded in the presence of blockers of AMPA and NMDA receptors. Mean base line membrane potential is -59mV (dashed line).
3. 25 Superimposed responses to a step current injection of variable amplitude (40-80pA, black), recorded from a single cell in the presence of synaptic blockers. Mean base line membrane potential is -59mV.

**Supplemental Fig. 2: DAP integral**

1. The DAP integral (mean±SEM) as a function of the injected current amplitude, summarizing 1309 stimuli given to 71 AOB mitral cells.
2. The DAP integral of 1309 stimuli given to 71 AOB mitral cells, plotted as a function of the firing frequency. The data is separated between cells that could be elicited to fire above 40 Hz (red) and those that could not (blue).

**Supplemental Fig. 3: Calcium imaging with and without BAPTA**

A-C) Mean somatic calcium indicator fluorescence signal (green) and membrane potential (gray) recorded during various firing patterns in a typical mitral cell (out of 5 recorded cells): a single spike (A); four spikes at 1Hz (B); forty spikes at 10Hz (C).

D) Mean somatic calcium indicator fluorescence signal with regular intracellular solution at 1Hz firing (red) and 10Hz firing (cyan) compared with the fluorescence signal recorded in the presence of 5mM intracellular BAPTA (n=3 cells) at 10 Hz firing (blue) and 30 Hz firing (green).
