# Supplementary figures and images for: Transient and sustained afterdepolarizations in accessory olfactory bulb mitral cells are mediated by distinct mechanisms that are differentially regulated by neuromodulators
Source: Front Cell Neurosci. 2015 Jan 14;8:432. doi: 10.3389/fncel.2014.00432 (PMC4294165; doi:10.3389/fncel.2014.00432)

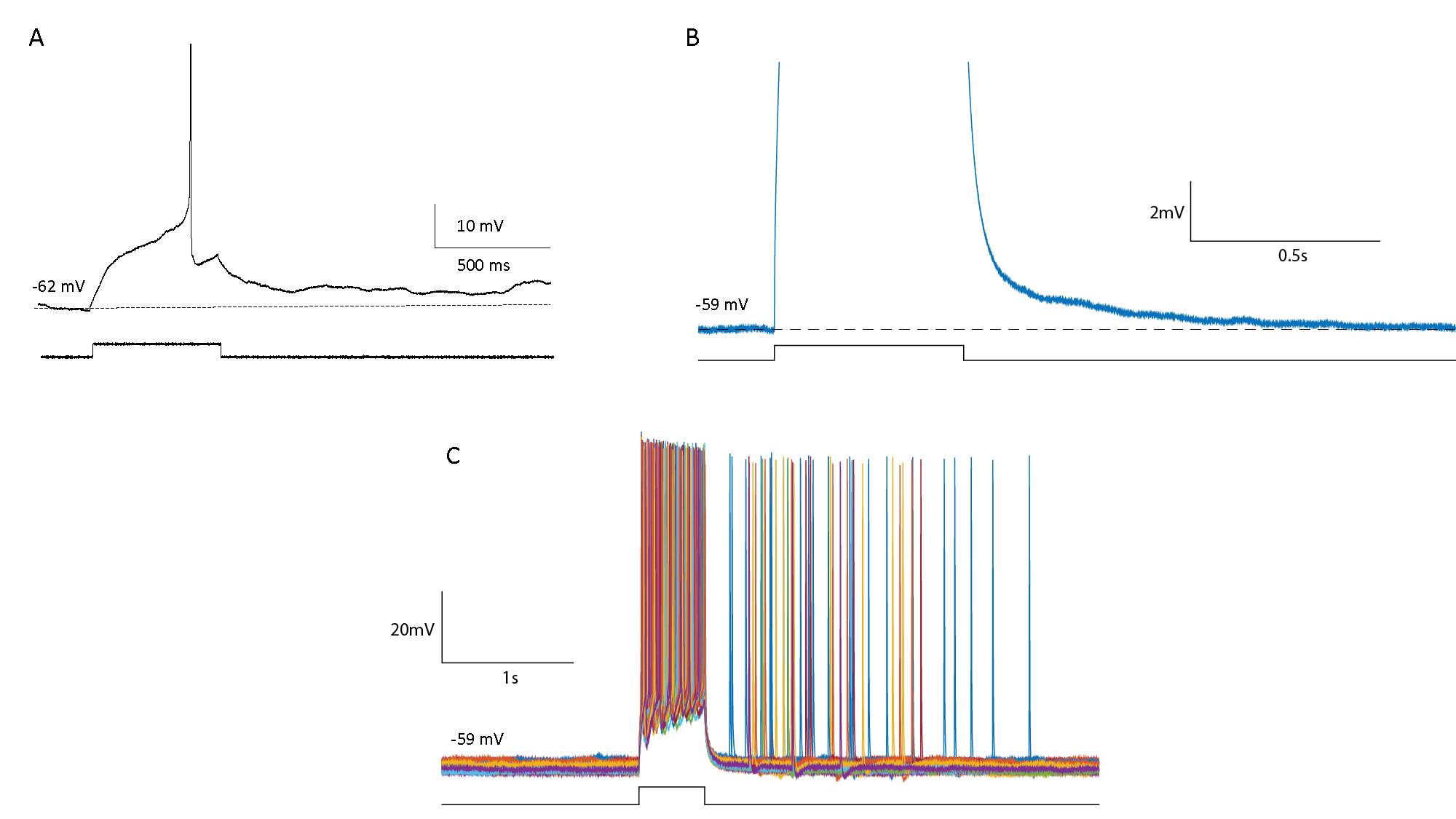

Supplement: Supplementary file 2 [file Image1.TIFF]

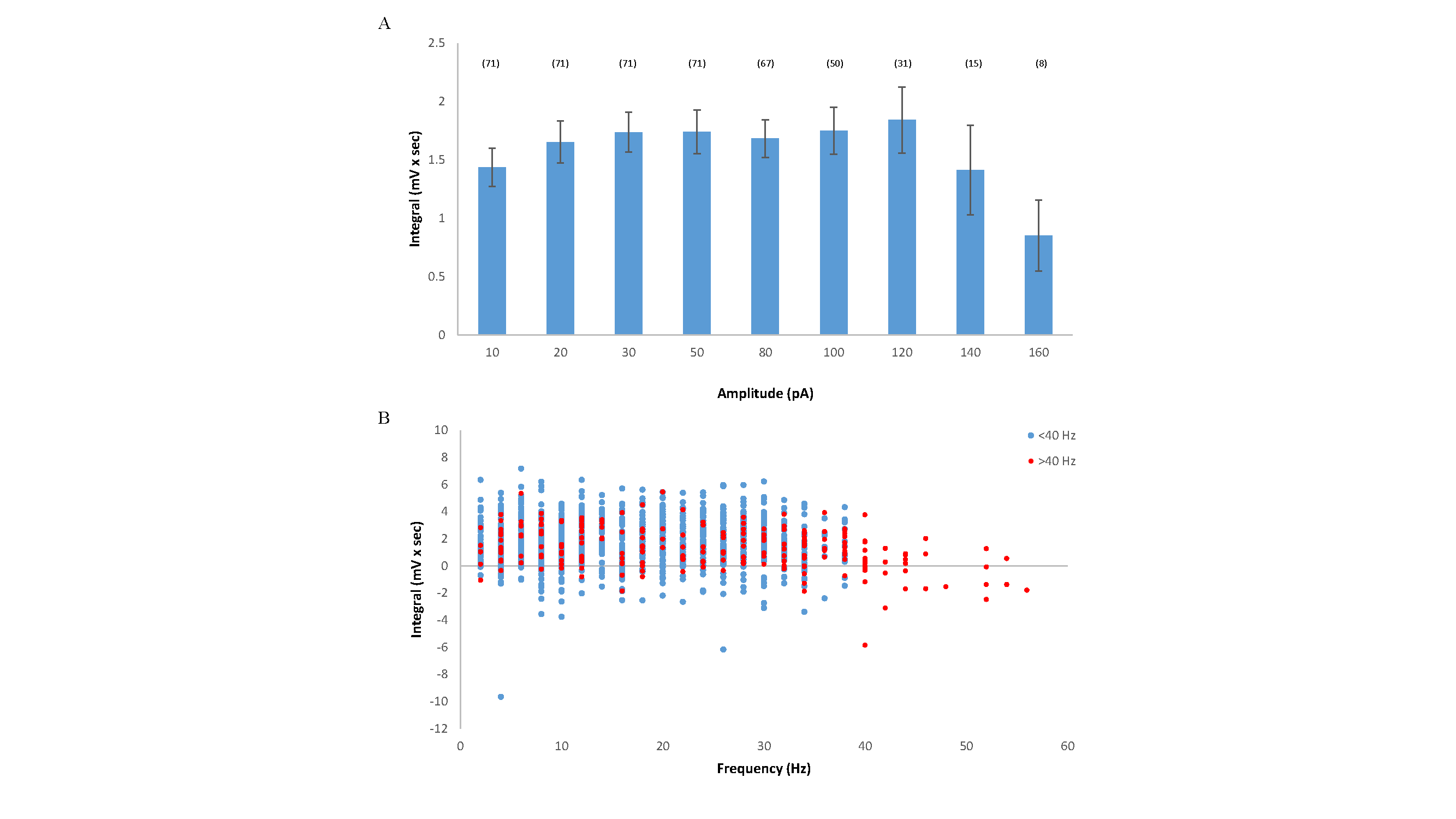

Supplement: Supplementary file 3 [file Image2.TIFF]

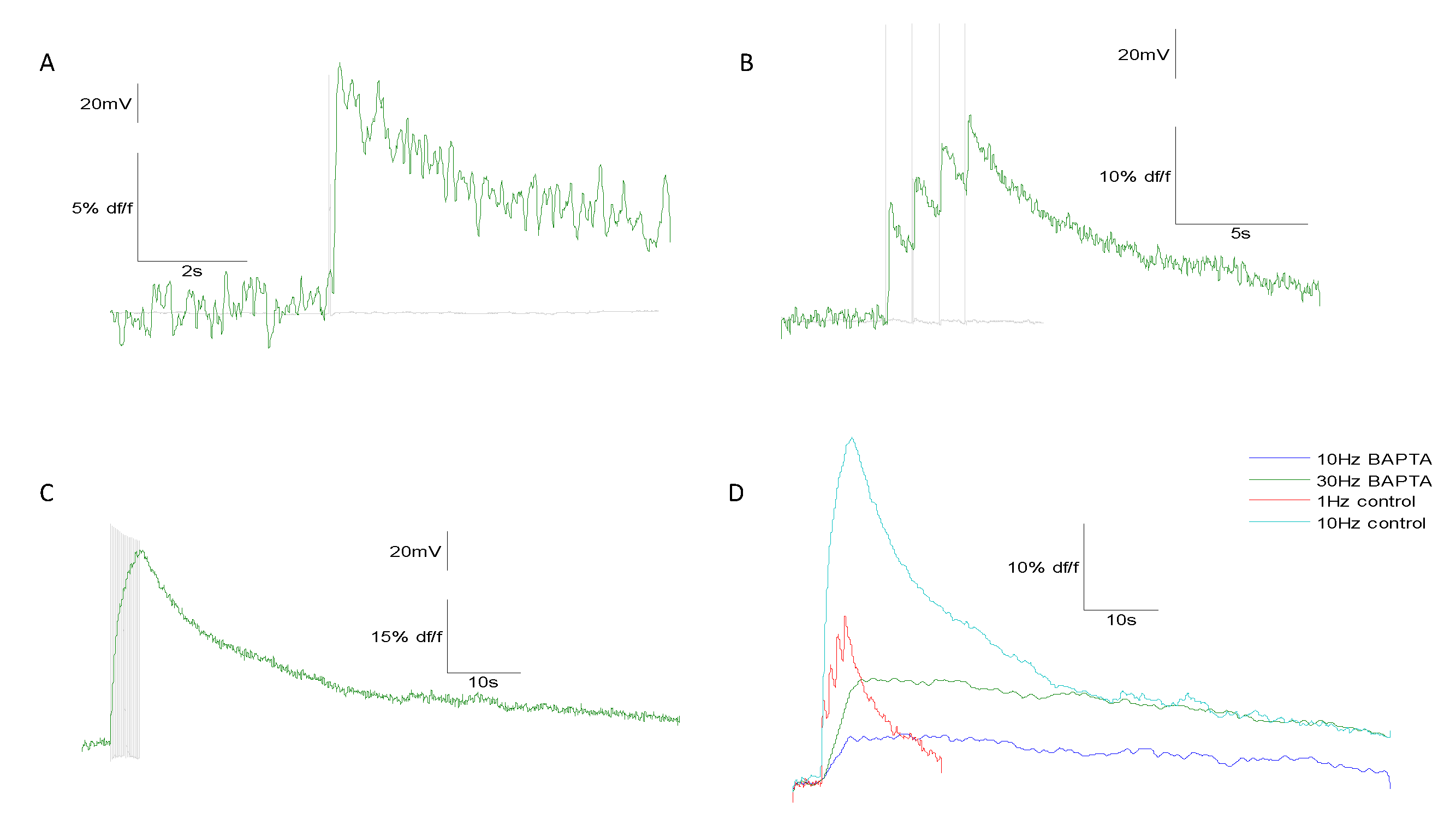

Supplement: Supplementary file 4 [file Image3.TIFF]
